# Prediction of extracapsular extension in prostate cancer using the Likert scale combined with clinical and pathological parameters

**DOI:** 10.3389/fonc.2023.1229552

**Published:** 2023-08-08

**Authors:** Jun-guang Wang, Bin-tian Huang, Li Huang, Xia Zhang, Pei-pei He, Jun-bo Chen

**Affiliations:** Department of Radiology, Ningbo Yinzhou No. 2 Hospital, Ningbo, Zhejiang, China

**Keywords:** prostate cancer, prostate-specific antigen, biopsy grade group, extracapsular extension, magnetic resonance imaging

## Abstract

**Abstract:**

This study aimed to investigate the independent clinical, pathological, and radiological factors associated with extracapsular extension in radical prostatectomy specimens and to improve the accuracy of predicting extracapsular extension of prostate cancer before surgery.

**Methods:**

From August 2018 to June 2023, the clinical and pathological data of 229 patients with confirmed prostate cancer underwent radical prostatectomy from The Second Hospital of Yinzhou. The patients’ multiparametric magnetic resonance imaging data were graded using the Likert scale. The chi-square or independent-sample T-test was used to analyze the related factors for an extracapsular extension. Multivariate analysis was used to identify independent factors associated with extracapsular extension in prostate cancer. Additionally, receiver operating characteristic curve analysis was used to calculate the area under the curve and assess the diagnostic performance of our model. The clinical decision curve was used to analyze the clinical net income of Likert scale, biopsy positive rate, biopsy GG, and combined mode.

**Results:**

Of the 229 patients, 52 had an extracapsular extension, and 177 did not. Multivariate analysis showed that the Likert scale score, biopsy grade group and biopsy positive rate were independent risk factors for extracapsular extension in prostate cancer. The area under the curves for the Likert scale score, biopsy grade group, and biopsy positive rate were 0.802, 0.762, and 0.796, respectively. Furthermore, there was no significant difference in the diagnostic efficiency for extracapsular extension (P>0.05). However, when these three factors were combined, the diagnostic efficiency was significantly improved, and the area under the curve increased to 0.905 (P<0.05). In the analysis of the decision curve, The clinical net income of the combined model is obviously higher than that of Likert scale, biopsy positive rate, and biopsy GG.

**Conclusion:**

The Likert scale, biopsy grade group and biopsy positive rate are independent risk factors for extracapsular extension in prostate cancer, and their combination can significantly improve the diagnostic efficiency for an extracapsular extension.

## 1 Introduction

Accurate local staging of prostate cancer plays a crucial role in prognosis and risk stratification. Prostate cancer with extracapsular extension (ECE) has a high rate of positive surgical margins, micrometastases, and biochemical recurrence (1).Approximately 27-36% of patients undergoing radical prostatectomy are found to have ECE (2). Preserving the neurovascular bundles (NVBs) and the dorsal vein complex (DVC) during surgery can significantly affect urinary and erectile function (3). Therefore, preoperatively identifying the site and extent of ECE helps formulate surgical plans and select appropriate patients (4), reducing the rate of positive surgical margins and increasing the number of patients undergoing NVB and DVC. Traditional methods to diagnose prostate cancer include serum prostate-specific antigen levels, digital rectal examination, and transrectal ultrasound-guided biopsy (5); however, the accuracy of these methods is limited (6).

Multiparametric magnetic resonance imaging (mpMRI) is the preferred method for assessing the local staging of prostate cancer, as it can accurately visualize the pelvic anatomy and assist in evaluating the involvement of NVB and DVC while identifying the location and extent of ECE (7). Nevertheless, non-structured reporting, such as indistinct or irregular margins of prostate capsule, can hinder communication between radiologists and urologists. Current prostate imaging reporting and data systems recommend using mpMRI for assessing local staging as the primary method, with ECE representing stage T3a and using the Likert scale for subjective assessment (8). However, current findings suggest that the accuracy of diagnosing ECE based on the Likert scale is limited (9). Combining clinical-pathological parameters with Likert scale data can identify independent factors related to ECEs’ diagnostic efficacy and provide a basis for urologists to formulate surgical plans.

## 2 Materials and methods

### 2.1 Study population

Clinical data were collected from 268 patients who underwent radical prostatectomy at The Second Hospital of Yinzhou from August 2018 to June 2023. Patients presenting with large MRI artifacts affecting diagnosis, those undergoing hormonal therapy, radiation/chemotherapy before MRI examination, and those with insufficient biopsy data were excluded. After these criteria were applied,229 patients were included in the current study.

### 2.2 MRI technique

All patients underwent mpMRI using a 1.5 T MRI (GESIGNA Voyager) scanner within 3 months before radical surgery. Scanning sequences included high-resolution T2-weighted imaging (TR4500 ms, TE110 ms), T1-weighted imaging (TR540 ms, TE15 ms), and diffusion-weighted imaging (TR6900 ms, TE100 ms, b = 1500 s/mm^2^). Dynamic contrast-enhanced T1WI (TR4.20 ms, TE1.70 ms) imaging was performed with 15 acquisitions, each lasting 11 seconds with a FOV of 24×24 cm and a slice thickness of 3 mm.

### 2.3 Image analysis

**Figure 1:**
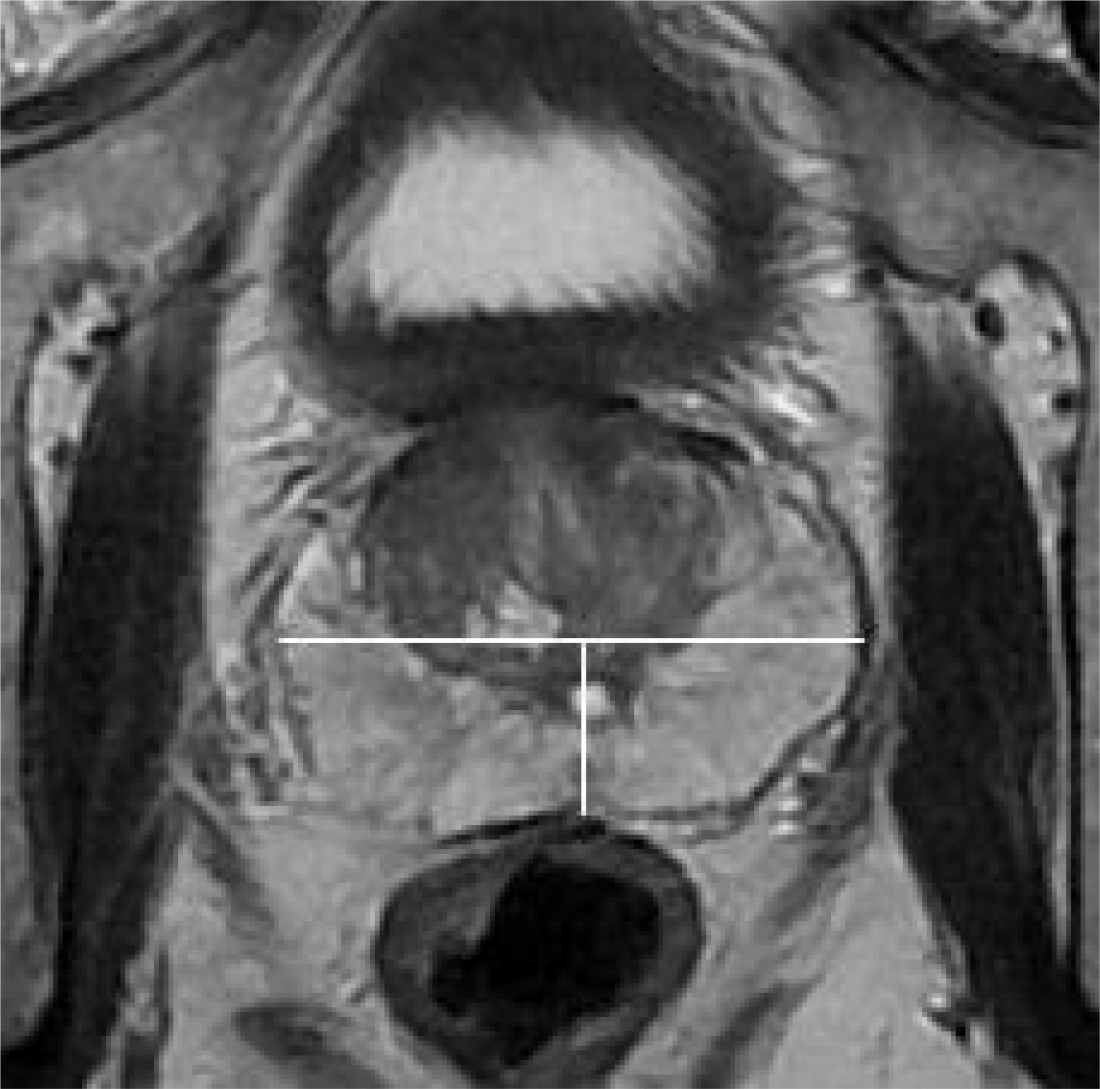
On axial T2WI, draw a straight line through the center of the prostate to divide it into anterior and posterior regions, and draw a vertical line to divide the posterior region into left and right posteriors(white line).

Experienced radiologists using the Likert scale without knowledge of biopsy results scored the probability of ECE: 1 point, no ECE; 2 points, unlikely ECE; 3 points, possible ECE; 4 points, probable ECE; and 5 points, highly probable ECE. Tumor locations were identified in the anterior, left, and right posterior regions (**Figure 1**).

### 2.4 Prostate biopsy and pathological analysis

**Figure 2:**
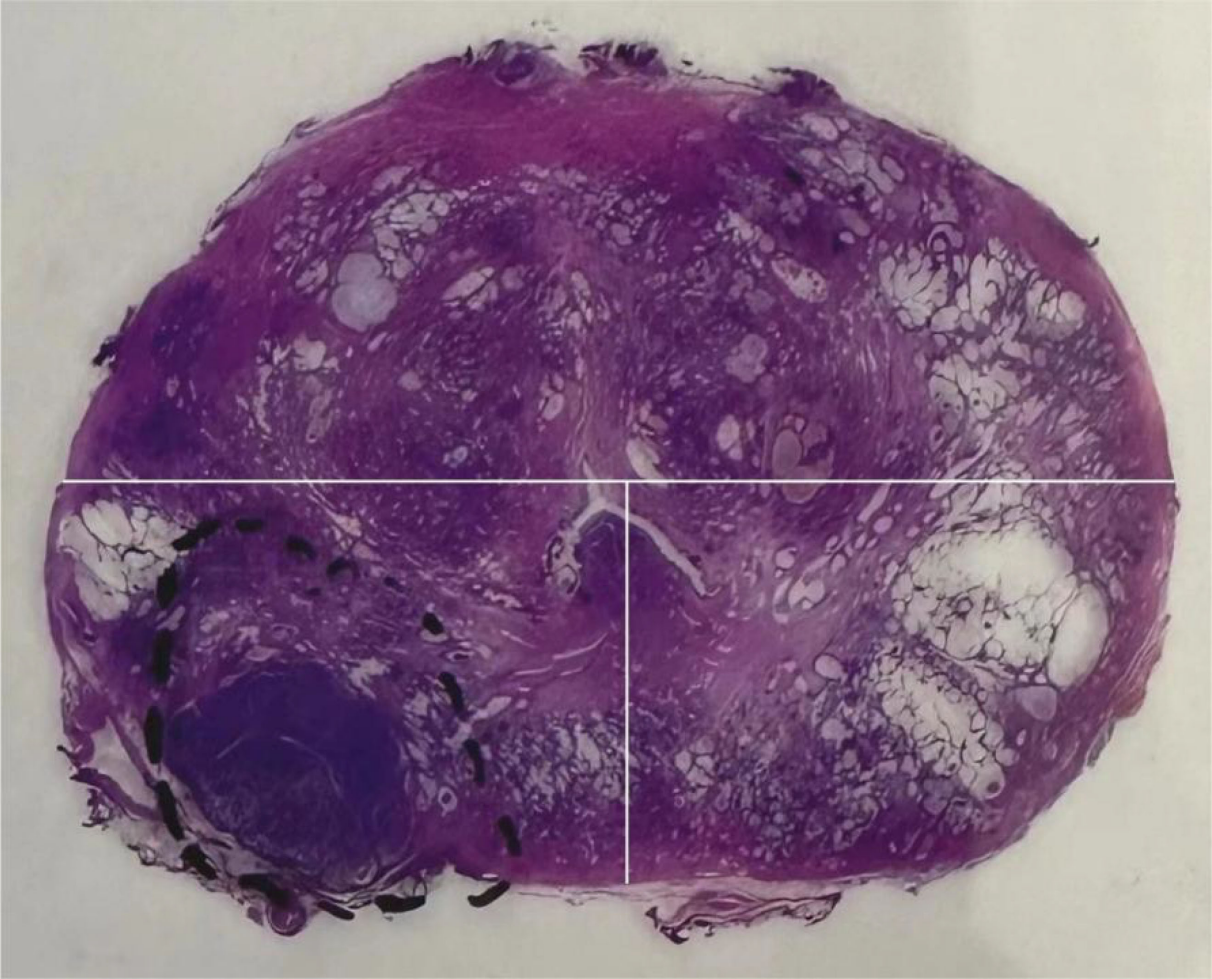
On the gross pathological specimen of radical prostatectomy, Divide the prostate into anterior, left posterior, and right posterior regions using the same division method as **Figure 1** (white line).

All patients underwent a standard systematic transrectal biopsy (12 cores) before surgery, with an additional 1-3 cores taken for lesions to be identified by mpMRI. ECE cases were defined as cancer cells extending beyond the prostate capsule into the surrounding adipose tissue. The location of ECE was determined from the gross pathology report, and tumor location was classified into anterior, left posterior, and right posterior locations according to the same approach used for MRI (**Figure 2**).

### 2.5 The variables

Clinical variables included age, prostate-specific antigen (PSA), biopsy positive rate, and biopsy grade group (GG) (Gleason score ≤6, 3 + 4, 4 + 3, 8, and 9-10, which corresponded to groups 1-5). MRI variables included the Likert scale score.

### 2.6 Statistical analysis

All statistical analyses were performed using SPSS (v17.0), MedCalc(v20.0), and Stata(v17.0)statistical software. Variables were compared using the chi-square or independent sample t-test. Multivariate logistic regression analysis was performed to determine independent risk factors for ECE, and receiver operating characteristic (ROC) curves were drawn for each independent factor and the combination of independent factors. The area under the curve (AUC) was calculated, and differences were compared using the DeLong test. A P-value less than 0.05 indicated statistical significance. drew the decision analysis curve of ECE for each independent factor and the combination of independent factors, by comparing the relative positions of each curve and the net profit rate corresponding to different risk thresholds (the incidence of ECE) and analysis the clinical benefits of ECE predicted by each independent factor and the combination of independent factors. Finally, a table was drawn to estimate the probability of ECE based on independent risk factors.

## 3 Results

### 3.1 Clinical and pathological outcomes

A total of 229 male patients were enrolled, of whom 52 had ECE. The mean patient age was 69.8 ± 6.0years, the mean PSA was 15.0 ± 13.3 ng/mL. There were 98 patients in biopsy GG 1-2, 99 in groups 3-4, and 32 in group 5. The mean biopsy positive rate was 43% (range: 7-100%) (**Table 1**).

**Table 1:** Characteristics of patients enrolled (n=229).

parameters	Value(Mean ± SD)
**Patient Age (y)**	69.8 ± 6.0
**PSA (ng/ml)**	15.0 ± 13.3
**Biopsy positive rate (%)**	0.43 ± 0.24
BiopsyGG n (%)	
**1**	46 (20.1)
**2**	52 (22.7)
**3**	45 (19.6)
**4**	54 (23.5)
**5**	32 (13.9)
Likert scale n (%)	
**1**	65 (28.3)
**2**	52 (22.7)
**3**	55 (24.0)
**4**	35 (15.2)
**5**	22 (9.6)

GG, grade group; PSA, prostate specific antigen.

### 3.2 MRI outcomes

mpMRI was performed in 182 patients 3 months before and 2 months after the biopsy in 47 patients. According to the Likert scale, 117 scored 1-2, 55 scored 3, 35 scored 4, and 22 scored 5 (**Table 1**).

### 3.3 Clinical pathological and MRI factors associated with ECE

ECE patients had significantly higher PSA and biopsy-positive rates than patients without ECE (24.9 [4.1-75.3] vs. 12.2[3.0-62.9] ng/mL, 66 [19-100] vs. 37 [7-82]%, respectively) (P<0.05). Biopsy GG and the Likert scale data were also significantly different between patients with and without ECE (P<0.05) (**Table 2**).

**Table 2:** Comparison of clinicopthological and mp-MRI factor.

	ECE +(n=52)	ECE -(n=177)	P-value
Clinicopathological			
**Patient Age**	71.3 ± 5.7	69.4 ± 6.0	0.06
**PSA (ng/ml)**	24.9 ± 17.1	12.2 ± 10.5	< 0.05
**Biopsy positive rate (%)**	66.1 ± 26.7	37.0 ± 20.1	< 0.05
**Biopsy GG n (%)**			< 0.05
**1-2**	5(9.6)	93(52.5)	
**3-4**	30 (57.6)	69(38.9)	
**5**	17 (32.6)	15 (8.4)	
mpMRI			
**Likert scale n (%)**			<0.05
**1-2**	7 (13.4)	110 (62.1)	
**3**	15(28.8)	40(22.5)	
**4**	17 (32.6)	18 (10.1)	
**5**	13 (25.0)	9 (5.1)	

ECE, extraprostatic extension; GG, grade group; PSA, prostate specific antigen.

Multivariate analysis showed that the biopsy-positive rate (P<0.05), biopsy GG (P<0.05), and Likert scores (P<0.05) were independent risk factors for ECE (**Table 3**).

**Table 3:** Multivariate analysis for predicting ECE using clinical and MRI parameters (n=229).

Variables	N	OR	95%CI	P-value
**Patient Age**	229	1.024	0.955-1.097	0.506
**PSA (ng/ml)**	229	1.029	0.992-1.068	0.127
**Biopsy positive rate (%)**	229	14.482	1.669-125.660	< 0.05
Biopsy GG n (%)				
**1-2**	98	ref.	ref	ref
**3-4**	99	9.694	2.465-38.126	< 0.05
**5**	32	12.162	2.714-54.489	< 0.05
Likert scale n (%)				
**1-2**	117	ref.	ref.	ref.
**3**	55	4.998	1.560-15.946	< 0.05
**4**	35	9.914	2.978-33.006	< 0.05
**5**	22	18.930	4.649-77.089	< 0.05

CI, confidence interval; ECE, extraprostatic extension; GG, grade group; OR, odds ratio; PSA, prostate specific antigen; ref, reference.

### 3.4 ROC analysis for ECE

**Figure 3:**
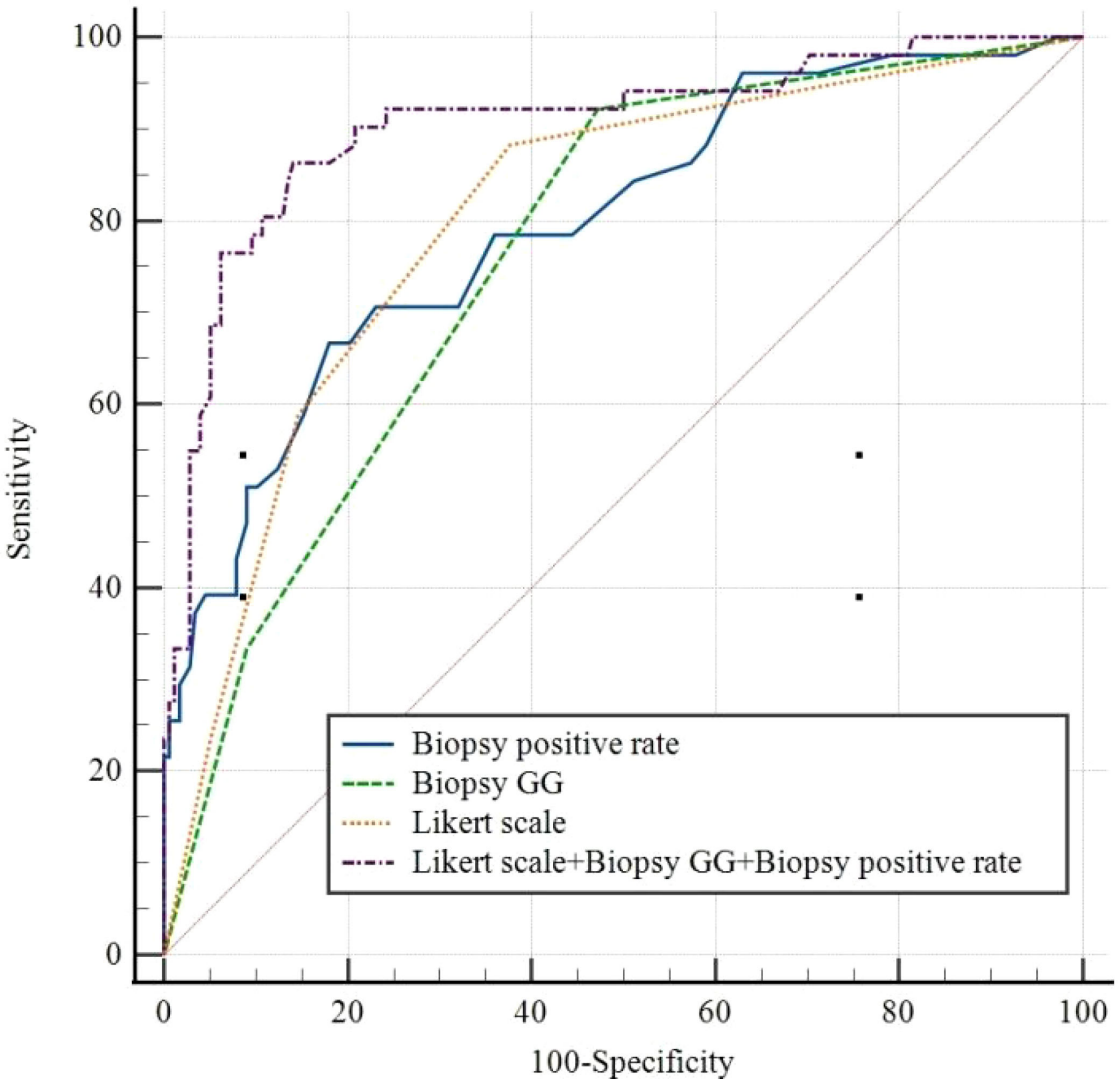
Receiver operating characteristic curves for Biopsy positive rate, Biopsy GG, Likert scale, and Likert scale+ Biopsy GG+ Biopsy positive rate. GG, grade group.

The ROC curves for each independent factor and the combination of independent factors showed that the AUC for biopsy positive rate, biopsy GG, and Likert scores were 0.796 (95% CI 0.738-0.847, P<0.05), 0.762 (95% CI 0.702-0.816, P<0.05), and 0.802 (95% CI 0.744-0.851, P<0.05), respectively (**Table 4**). Additionally, the AUC for the combination of independent factors was 0.905 (95% CI 0.859-0.939, P<0.05) (**Figure 3**; **Table 4**).

**Table 4:** ROC analysis for ECE.

	AUC	95%CI	P-value
Clinicopathological			
**Biopsy positive rate**	0.796	0.738-0.847	<0.05
**Biopsy GG**	0.762	0.702-0.816	<0.05
mpMRI			
**Likert scale**	0.802	0.744-0.851	<0.05
**Likert scale+ Biopsy GG+ Biopsy positive rate**	0.905	0.859-0.939	<0.05

AUC, area under curve; CI, confidence interval; ECE, extraprostatic extension; GG, grade group; mpMRI, multiparameter magnetic resonance imaging.

Notably, there was no statistically significant difference in the diagnostic performance for ECE among biopsy positive rate, biopsy GG, and Likert scores (P>0.05). However, the combination of these indicators showed significantly higher diagnostic performance than any single one (P<0.05) (**Table 5**).

**Table 5:** Comparison of AUC value in different ECE prediction schemes.

	Biopsy positive rate	Biopsy GG	Likert scale	Likert scale+ Biopsy GG+ Biopsy positive rate
Percent positive cores				
**Biopsy GG**	0.372			
**Likert scale**	0.917	0.346		
**Likert scale+ Biopsy GG+ Biopsy positive rate**	<0.05	<0.05	<0.05	

AUC, area under curve; ECE, extraprostatic extension; GG, grade group.

### 3.5 Clinical decision curves of the biopsy positive rate, biopsy GG, Likert scores and combined model

**Figure 4:**
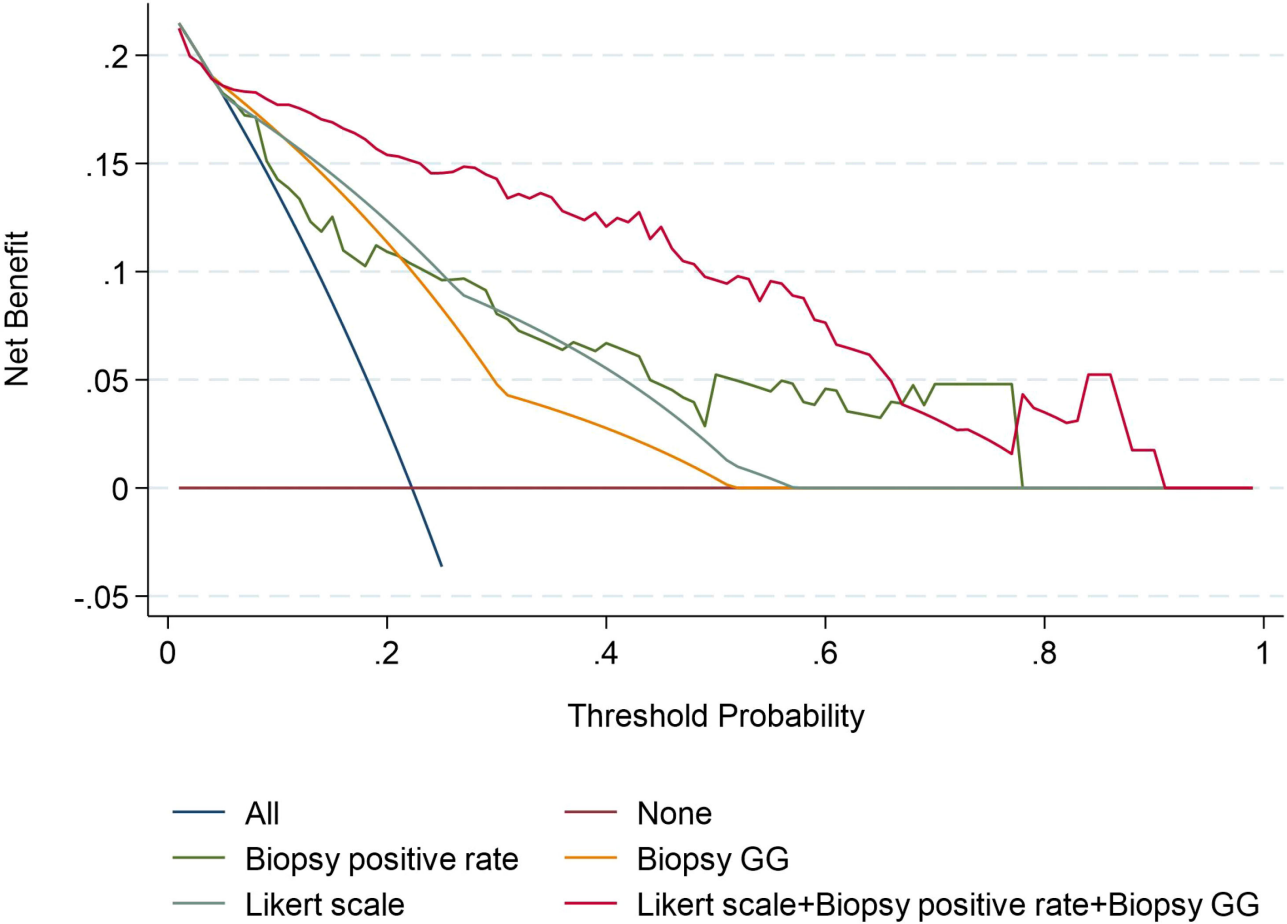
Decision curves of the Biopsy positive rate, Biopsy GG, Likert scale, and Likert scale+ Biopsy GG+ Biopsy positive rate model for diagnosing ECE. GG, grade group.

In the analysis of the clinical decision curve, at different risk thresholds, the analytical curve of biopsy positive rate, biopsy GG, Likert scores and Combined Model predicting ECE were located at the upper right of the two extreme curves, indicating that they all had higher net benefits. At most risk thresholds, the net benefit of the combine model was significantly higher than that of the biopsy positive rate, biopsy GG, and Likert scores (**Figure 4**).

### 3.6 Probability of ECE stratified by the biopsy positive rate, the biopsy GG, and the Likert scale score

A combination of biopsy positive rate (<49% vs. ≥49%), biopsy GG (1-2 vs. 3-4 vs. 5), and Likert scale score data (1-2 vs. 3 vs. 4 vs. 5) was used to predict the probability of ECE in prostate cancer. When the biopsy positive rate was <49%, the risk of ECE was low (8%, 12/155), regardless of the biopsy GG and Likert scale score. When the biopsy positive rate was ≥49%, the risk of ECE was higher with Likert scale scores of 3-5 in conjunction with biopsy GG 3-5 (70%-100%) than with Likert scale scores of 1-2 (9%-40%) or biopsy GG 1-2 (0%-50%) (**Table 6**).

**Table 6:** Probability of ECE stratified with Biopsy positive rate, biopsy GG, and Likert scale.

Biopsy positive rate (%)		<49, n (%)			≥49, n (%)			Total
Biopsy GG		1-2	3-4	5	1-2	3-4	5	
Likert scale	1-2	0/56 (0%)	1/33 (3%)	0/2 (0%)	1/11 (9%)	4/10 (40%)	1/5 (20%)	7/117 (6%)
	3	0/18 (0%)	3/17 (17%)	0/2 (0%)	0/1 (0%)	7/10 (70%)	5/7 (71%)	15/55 (27%)
	4	0/4 (0%)	2/10 (20%)	1/2 (50%)	2/4 (50%)	5/6 (83%)	7/9 (77%)	17/35 (49%)
	5	1/2 (50%)	2/5 (40%)	2/4 (50%)	1/2 (50%)	6/8 (75%)	1/1 (100%)	13/22 (59%)
Total		1/80 (1%)	8/65 (12%)	3/10 (30%)	4/18 (22%)	22/34 (65%)	14/22 (64%)	52/229 (23%)

Date are shown by “the number of cases with ECE (+)/total cases” and the percentage. The white, blue, and orange areas indicate the low (<40%), intermediate (40-59) and high (≥60%) probability of ECE. ECE, extraprostatic extension; GG, grade group.

### 3.7 MRI and ECE tumor co-location

Out of 229 patients, 205 had a corresponding tumor location in both mpMRI images and pathological specimens, with a matching percentage of 89%. The percentages for the anterior, left posterior, and right posterior regions were 88% (97/109), 89% (53/59), and 90% (55/61), respectively. Of the 52 patients with ECE, 48 had corresponding locations of tumor ECE in both the MRI images and pathological specimens, with a matching percentage of 92%. The percentages for the anterior, left posterior, and right posterior regions were 94% (16/17), 90% (18/20), and 93% (14/15).

## 4 Discussion

This study uses preoperative clinicopathological data and the Likert scale based on mpMRI to assess ECE risk in prostate cancer. Our findings suggest that the biopsy positive rate, biopsy GG, and the Likert score are independent risk factors for ECE in prostate cancer. Combining these independent risk factors can improve the diagnostic accuracy of ECE in prostate cancer.

Clinical pathological factors have high accuracy in predicting ECE in prostate cancer. Biopsy positive rate (AUC = 0.79) and have higher accuracy in predicting ECE in prostate cancer than biopsy GG (AUC = 0.76); however, this difference is not statistically significant (P>0.05). In previous studies, biopsy GG was reported to be an independent risk factor for ECE in prostate cancer (10), and the AUC for prediction of ECE was 0.68 to 0.71 (11, 12), slightly lower than that of our study (0.76). Additionally, the AUC for the prediction of ECE using a biopsy positive rate was 0.77 (13), slightly lower than that of our study (0.79). Differences in patient race, biopsy method, and the total number of biopsy needles may have contributed to the differences in the AUC observed in this study.

mpMRI has been found to have high specificity but low sensitivity in detecting ECE in prostate cancer (14, 15). Multiple structured evaluation methods exist for studying ECE in prostate cancer, including EPE grading, the Likert scale and PI-RADS (16). EPE grading,Likert scale, and PI-RADS in predicting ECE had good diagnosis effect (0.77, 0.78, 0.73, respectively) (17, 18). Onay et al. refined the Likert scale to predict ECE of prostate cancer (19). Wibmer et al. study showed that, the Likert grading standardized reporting system can significantly improve diagnostic accuracy of predicting ECE (20). Recent study showed that the Likert scale had significant diagnostic efficacy in predicting biochemical recurrence and lymph node metastasis of prostate cancer (21). Subsequently, some scholars conducted external verification on Likert scale to predict ECE of prostate cancer, and the results showed that Likert scale had high accuracy in predicting ECE of prostate cancer(sensitivity 0.47, specificity 0.90, respectively) (22). Recent studies suggest that the Likert scale is an independent risk factor for ECE and can better predict it (23). The AUC values for the Likert scale were found to be 0.86 (24), higher than our study’s value of 0.80. We hypothesize that this difference could be due to the inclusion of patients who underwent mpMRI after biopsy, which could have caused tissue proliferation and inflammation leading to irregular or thickened capsule morphology, resulting in higher Likert scores.

Combining mpMRI images with clinical and pathological indicators can improve the accuracy of predicting ECE. Recent studies have shown that the AUC value for diagnosing ECE using the Likert scale alone was 0.78 while combining the Likert scale with biopsy GG resulted in an AUC value of 0.85 (25, 26). Our study’s results are consistent with these findings, with the AUC values for biopsy positive rate, biopsy GG, and Likert scale alone being 0.796, 0.762, and 0.802, respectively. Combining all these factors resulted in an AUC value of 0.905. After the combination of Likert scale and clinical indicators, the combined model can obtain higher clinical benefits. on this basis one study showed that the PI-RADS score combined with clinical indicators had high diagnostic power in predicting lymph node metastasis in prostate cancer (27). The clinical decision curve also showed that the combined mode had a higher net benefit than Likert scale, biopsy positive rate, and biopsy GG, the clinical benefits was significantly improved, and Likert scale can provide the location of pathological ECE, improve the accuracy of clinical evaluation of prostate cancer.

When the biopsy positive rate was ≥49%, the risk of ECE was high (76%, 31/41) for those who scored 3-5 on the Likert scale and belonged to biopsy GG 3-5. The risk of ECE was low (27%, 9/33) for those who scored 1-2 on the Likert scale or belonged to biopsy GG 1-2. When the biopsy positive rate was <49%, the risk of ECE was low (8%, 12/155) regardless of the Likert scale score or biopsy GG. This information can help estimate the probability of ECE in prostate cancer.

The correspondence rate between the location of ECE in histopathological specimens and mpMRI is 89%, providing evidence-based guidance for urologists to decide whether to preserve or excise NVBs and DVC during surgery for prostate cancer.

This study has some limitations. First, the retrospective design may lead to selection bias. Second, 20% of patients underwent mpMRI after biopsy, which could affect the Likert score. Lastly, variations in surgical approaches by urologists may influence pathologists’ evaluation of ECE and may affect the conclusions made in the current study.

## 5 Conclusion

In summary, the biopsy-positive rate, biopsy GG, and Likert scale score are independent risk factors for ECE in prostate cancer, and the combined assessment of these parameters improves the accuracy of predicting ECE, guiding urologists in surgical planning.

## Data availability statement

The raw data supporting the conclusions of this article will be made available by the authors, without undue reservation.

## Ethics statement

The studies involving human participants were reviewed and approved by the Ethic Committee of Ningbo Yinzhou No. 2 Hospital. Written informed consent to participate in this study was provided by the participants OR patients.

## Author contributions

J-GW was responsible for study design, data acquisition and analysis, and manuscript writing. J-GW and B-TH performed bioinformatics and statistical analyses. J-GW, LH, and P-PH were responsible for collecting clinical samples. J-GW and XZ prepared the figures and tables for the manuscript. J-BC was responsible for the integrity of the entire study and manuscript review. All authors contributed to the article and approved the submitted version.
